# Supplementary material for: Locomotion Outcome Improvement in Mice with Glioblastoma Multiforme after Treatment with Anastrozole
Source: Brain Sci. 2023 Mar 15;13(3):496. doi: 10.3390/brainsci13030496 (PMC10046174; doi:10.3390/brainsci13030496)
Supplement: Supplementary file 1 [file brainsci-13-00496-s001.zip › brainsci-2270366-supplementary.pdf]

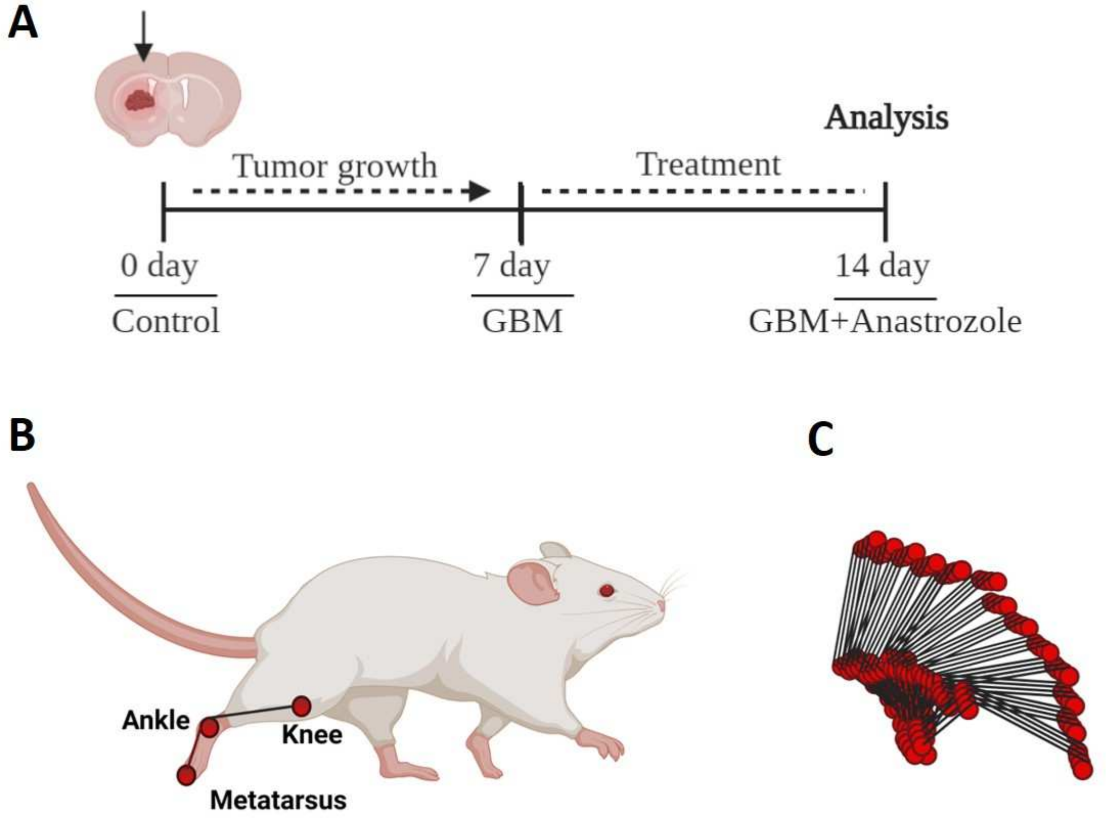

Figure S1. Schematic diagram shows the experimental approach. A) a coronal section exhibiting xenograft localization (As indicated by the arrow). Day 0 indicates the onset of the experiment. Day 7 indicated tumor growth and the onset of anastrozole treatment. Day 14 indicates when the anastrozole treatment did finish. B) Parameters evaluated for gait locomotion. The articular red joint points considered were the metatarsus, ankle, and knee. C) Schematic representation of the evaluated displacement curves of articular joints calculated using the Euclidean distance (see Methods).
